# Age at dementia diagnosis, subtype associations, and neuroimaging differentiation in Bangladesh: The CARED study

**DOI:** 10.1371/journal.pone.0359109

**Published:** 2026-09-25

**Authors:** Redoy Ranjan, Ghulam Kawnayn, Md. Abdullah Yusuf, Mohammad Nur Uddin, Mim Tanzila Mamun, Alif Al Mamun, Shadad Hossain, Mohammad Sayeed Hassan, Nafiz Imtiaz, Maliha Hakim

**Affiliations:** 1 Department of Cardiac Surgery, Bangabandhu Sheikh Mujib Medical University, Dhaka, Bangladesh; 2 Department of Biological Sciences, Royal Holloway University of London, London, United Kingdom; 3 Department of Neurology, Combined Military Hospital, Chittagong, Bangladesh; 4 Department of Microbiology, National Institute of Neurosciences and Hospital, Dhaka, Bangladesh; 5 Department of Neurology, National Institute of Neurosciences and Hospital, Dhaka, Bangladesh; 6 Department of Medicine, Chottogram Army Medical College, Chittagong, Bangladesh; 7 Department of Neuroscience, Macquarie University, Sydney, Australia; 8 Department of Education, Southern Cross University, Sydney, Australia; 9 Department of Psychiatry, North East London NHS Foundation Trust, London, United Kingdom; 10 Department of Psychiatry, Aqidah Healthcare, Dhaka, Bangladesh; University of Thessaly Faculty of Medicine: Panepistemio Thessalias Tmema Iatrikes, GREECE

## Abstract

**Background:**

Evidence on age at diagnosis of dementia and differentiation of dementia subtypes in Bangladesh is limited. This study aimed to determine age at diagnosis, examine factors associated with dementia subtypes, and evaluate the discriminatory utility of neuroimaging scales in a Bangladeshi clinical population.

**Methods:**

The Community Awareness and Research on Early Dementia (CARED) study was a cross-sectional observational study conducted 2019−2024 at a tertiary care centre in Bangladesh. A total of 658 adults presenting with cognitive impairment, undergoing evaluation for suspected dementia were included. Diagnoses were established using DSM-5 criteria by a multidisciplinary team. Patients were categorized into dementia subtypes. Pseudodementia was retained as separate comparator group. Multivariable regression analyses were performed to identify factors associated with dementia subtypes. Receiver operating characteristic (ROC) curve analyses were used to assess the discriminatory performance of neuroimaging scales.

**Results:**

Among 658 patients, Alzheimer’s-related dementia (ARD) accounted for 51.7% of cases. The mean age at diagnosis was significantly higher in ARD compared with non-Alzheimer’s-related dementia (nARD) (67.7 ± 10.8 vs 64.3 ± 11.4 years, P < 0.001). In multivariable analysis, nARD was associated with an earlier age at diagnosis by 4.3 years compared with ARD. Vascular dementia was associated with hypertension, OR 4.2 (95% CI 1.1–16.6), P = 0.04, and smoking, OR 13.1 (95% CI 1.6–103.3), P = 0.01. Neuroimaging scales demonstrated variable discriminatory performance. Medial temporal atrophy showed good discrimination for ARD (AUROC 0.752), while Fazekas scores demonstrated good performance for vascular dementia (AUROC 0.838) and mixed dementia (AUROC 0.836). Global cortical atrophy showed moderate discriminatory ability for frontotemporal dementia (AUROC 0.659).

**Conclusions:**

Age at diagnosis varied across dementia subtypes in this Bangladeshi clinical cohort, with non-Alzheimer’s diagnosed earlier than Alzheimer’s. Structured MRI visual rating scales showed variable subtype-specific discriminatory performance, suggesting that they may provide adjunctive information for dementia subtype differentiation when interpreted alongside multidisciplinary clinical assessment.

## Introduction

Dementia is a broad term for progressive cognitive impairment that interferes with daily functioning. Around 10 million new cases are reported globally each year, primarily among older adults [1,2]. The estimated number of people living with dementia is around 55 million globally and is expected to triple by 2050 due to ageing populations [2,3]. Dementia is a multifactorial complex disease whose common risk factors include advanced age, family history, genetic predispositions such as the presence of the APOE ε4 allele, lower levels of education, head trauma and lifestyle factors like smoking, physical inactivity, and poor diet [4,5]. Further, chronic conditions such as diabetes, hypertension, obesity, and cardiovascular disease also increase the likelihood of developing dementia [6–8]. Alzheimer’s disease is the most common cause of dementia, followed by vascular dementia and frontotemporal dementia [9]. The pathology of dementia varies, especially Alzheimer’s-related dementia (ARD), characterised by amyloid-beta plaques and tau protein tangles, leading to neuronal death, while non-Alzheimer’s-related dementia (nARD) results from cerebrovascular damage and neurodegeneration [10,11].

Dementia primarily affects adults aged 65 and older [12,13]; though rare, early-onset dementia can occur before this age [14]. Consequences of dementia include progressive loss of independence, malnutrition, dehydration, infections like pneumonia, and behavioural and psychological symptoms such as agitation, depression, and psychosis, which also contribute to caregiver burden [15–17]. Late-stage dementia may result in total dependency, incontinence, and immobility. Dementia is progressive and incurable; prognosis varies by type and severity, with an average survival of 5–10 years after diagnosis, though some may live longer [18,19]. Early diagnosis and intervention can improve quality of life, while palliative care is essential in advanced stages to manage symptoms and maintain dignity [3,5,18].

Radiological investigations like structural magnetic resonance imaging (MRI) play an important role in the evaluation of patients with cognitive impairment by helping exclude reversible structural causes and supporting differentiation between dementia subtypes. Practical visual rating scales, including medial temporal atrophy, global cortical atrophy, Koedam, and Fazekas scores, may provide accessible tools for subtype assessment, particularly in resource-limited settings where advanced biomarker testing is less available [20].

This study aimed to determine the age at diagnosis across dementia subtypes in a Bangladeshi clinical population, examine factors associated with different dementia subtypes, and evaluate the discriminatory utility of different neuroimaging scoring systems across dementia subtypes.

## Patients and methods

### Study design and setting

The Community Awareness and Research on Early Dementia (CARED) study is a cross-sectional observational study conducted at the National Institute of Neurosciences and Hospital (NINS&H), Dhaka, Bangladesh, between 1 January 2019 and 31 December 2024. The study data were accessed for research purposes on 30 May 2025. All eligible individuals aged 40 years and older presenting with subjective cognitive complaints and undergoing evaluation for suspected dementia during the study period were consecutively included; therefore, no a priori sample-size calculation was performed. Ethical approval was obtained from the National Institute of Neurosciences and Hospital Ethics Committee, Dhaka, Bangladesh (IRB/NINS/2024/393). Written informed consent was obtained from all participants before enrolment.

### Clinical assessment and diagnostic procedure

All participants underwent a comprehensive clinical evaluation, including detailed history taking and neurological examination conducted by a neurologist. Diagnosis of dementia was established through a multidisciplinary team (MDT) approach involving neurologists, neuroradiologists, and psychiatrists. Dementia diagnoses were confirmed according to the Diagnostic and Statistical Manual of Mental Disorders, Fifth Edition (DSM-5) criteria [21]. A Bangla-adapted version of the Mini-Mental State Examination (MMSE) was administered as part of the objective cognitive assessment to support the diagnosis of dementia during routine clinical evaluation [22]. Patients with cognitive symptoms attributable to psychiatric disorders, including pseudodementia, were identified through MDT assessment and excluded from the neurodegenerative dementia group. These patients were retained as a separate comparator group for analysis. To minimise diagnostic misclassification, non-neurodegenerative structural causes of cognitive impairment (e.g., intracranial tumours, infections, head injury, and metabolic abnormalities) were excluded through clinical and imaging evaluation.

### Neuroimaging assessment

All participants underwent magnetic resonance imaging (MRI) using Siemens 1.5T and 3T Magnetom Symphony systems, following the hospital imaging protocol. Imaging was performed at the Department of Neuroradiology and Imaging, NINS&H.

Neuroimaging was evaluated using established visual rating scales, including medial temporal atrophy (MTA) [23,24], global cortical atrophy (GCA) [25], Koedam score [26], and Fazekas (Fz) [27] score. Ratings were performed by neuroradiologists blinded to clinical findings and patient age. Final imaging scores were assigned following MDT consensus.

### Data collection

Demographic and clinical data, including age, sex, residence, and vascular risk factors (hypertension, diabetes mellitus, smoking, chronic kidney disease, and family history of dementia), were collected from patients, caregivers, and medical records using standardised procedures.

To exclude reversible causes of cognitive impairment, laboratory investigations were performed, including blood glucose, serum creatinine, complete blood count, peripheral blood film, thyroid function tests (TSH, FT3, FT4), vitamin B12, vitamin D, calcium, parathyroid hormone, liver function tests, and infectious screening (VDRL, TPHA). Chest radiography was also performed where indicated.

### Statistical analysis

Statistical analyses were performed using SPSS version 28.0. Univariate analyses were initially conducted to explore associations between clinical variables and dementia subtypes. Variables with P ≤ 0.10 were entered into stepwise multivariable logistic regression models using a backward elimination approach to identify factors associated with different dementia subtypes.

To evaluate the discriminatory utility of neuroimaging scales across dementia subtypes, receiver operating characteristic (ROC) curve analyses were performed. The 95% confidence intervals for the AUROC estimates were calculated using the nonparametric asymptotic method implemented in SPSS. Optimal cut-off values were determined using the Youden index. Model performance was assessed using the area under the receiver operating characteristic curve (AUROC), along with sensitivity, specificity, positive predictive value (PPV), and negative predictive value (NPV).

Missing data were assessed using Little’s Missing Completely at Random (MCAR) test. Multicollinearity among independent variables was evaluated using variance inflation factors (VIFs) and tolerance values, with VIF > 10 or tolerance < 0.1 indicating significant multicollinearity [28]. A two-tailed P value ≤ 0.05 was considered statistically significant. No adjustment was made for multiple comparisons because the subtype analyses were exploratory.

## Results

A total of 658 patients presenting with cognitive impairment were evaluated, of whom ARD accounted for 340 cases (51.7%) and nARD for 318 cases (48.3%). The cohort was classified into neurodegenerative dementia subtypes and pseudodementia, which was retained as a comparator group. The cohort was predominantly male (60.3%), with an overall age range of 41–83 years. Baseline characteristics stratified by sex are provided in S1 Table. The mean age at diagnosis was significantly higher in ARD compared with nARD (67.7 ± 10.8 vs 64.3 ± 11.4 years, P < 0.001). MTA and Koedam scores were significantly higher in ARD, whereas Fazekas scores were significantly higher in nARD. No significant differences were observed for sex, residence, hypertension, diabetes, depression, smoking, chronic kidney disease, family history of dementia, TSH, FT4, or GCA score (Table 1).

**Table 1:** Baseline characteristics of study population (N = 658).

Variables		Alzheimer’s-related Dementia (n = 340)	Non-Alzheimer’s-Related Dementia (n = 318)	P value
Age (years, mean ± SD)		67.7 ± 10.8	64.3 ± 11.4	<0.001
Male, n (%)		193 (49.0%)	201 (51.0%)	0.13
Residence, n (%)	Rural	95 (55.2%)	77 (44.8%)	0.63
	Urban	113 (55.4%)	91 (44.6%)	
	Town	75 (50.7%)	73 (49.3%)	
HTN, n (%)		160 (49.5%)	163 (50.5%)	0.21
Diabetes, n (%)		112 (51.9%)	104 (48.1%)	0.96
Depression, n (%)		138 (53.7%)	119 (46.3%)	0.19
Smoking, n (%)		54 (52.4%)	49 (47.6%)	0.55
CKD, n (%)		16 (53.3%)	14 (46.7%)	0.61
Familial dementia, n (%)		48 (58.5%)	34 (41.5%)	0.17
MRF (≥2RF), n (%)		168 (52.3%)	153 (47.7%)	0.85
TSH (mean ± SD)		2.1 ± 1.6	2.3 ± 1.8	0.34
FT4 (mean ± SD)		2.0 ± 3.3	1.4 ± 1.8	0.10
Koedam score (mean ± SD)		1.1 ± 0.8	0.8 ± 0.5	<0.001
MTA score (mean ± SD)		2.1 ± 0.7	1.4 ± 0.6	<0.001
GCA score (mean ± SD)		1.2 ± 0.5	1.2 ± 0.5	0.60
Fazekas score (mean ± SD)		1.0 ± 0.5	1.6 ± 0.7	<0.001

Data are presented as mean ± SD for continuous variables and n (row %) for categorical variables. Independent-samples t tests were used for continuous variables, and chi-square tests were used for categorical variables. P values compare Alzheimer’s-related dementia with non-Alzheimer’s-related dementia. A two-tailed P value < 0.05 was considered statistically significant. ARD = Alzheimer’s-related dementia; nARD = non-Alzheimer’s-related dementia; HTN = hypertension; CKD = chronic kidney disease; MRF = multiple risk factors; RF = risk factor; TSH = thyroid-stimulating hormone; FT4 = free thyroxine; MTA = medial temporal atrophy; GCA = global cortical atrophy.

In multivariable linear regression analysis (Table 2), dementia subtype remained independently associated with age at diagnosis, with nARD diagnosed on average 4.3 years earlier than ARD (β = −4.3, SE = 2.0, P = 0.03). Higher neuroimaging scores were also associated with older age at diagnosis, including MTA (β = 5.3, P < 0.001), Koedam (β = 2.2, P < 0.001), GCA (β = 2.3, P = 0.007), and Fazekas (β = 2.0, P = 0.002). Although statistically significant, the model explained only a small proportion of the variability in age at diagnosis (R² = 0.03) (Table 2).

**Table 2:** Stepwise multivariable linear regression analysis predicting age of dementia diagnosis (years) with risk factors.

	Effect size (*β*), years	SE	P value	95% CI		F test	R^2^
				Lower	Upper		
Constant	69.3	1.3	<0.001	66.7	71.7	–	–
Dementia group: nARD versus ARD	−4.3	2.0	0.03	−8.3	−0.4	4.6	0.03
MTA	5.3	0.5	<0.001	4.2	6.4	90.9	0.122
Koedam	2.2	0.6	<0.001	1.1	3.3	14.5	0.022
GCA	2.3	0.8	0.007	0.6	4.0	7.2	0.011
Fazekas	2.0	0.6	0.002	0.7	3.2	9.6	0.015

Dependent Variable: Age

Variables Entered into Model: Sex, Residence, Depression, HTN, DM, TSH, FT4, Family H/O dementia, Koedam, MTA, GCA, and Fazekas score. Only variables retained after stepwise selection are shown in the final model.

Effect size (β-coefficients), degree of change in the age of dementia diagnosis (dependent variable) for each one-unit of change in the predictor variable. The significance of the F-test value indicates a linear relationship between the variables in the model. R2 value is the percentage of variance in age of dementia diagnosis explained by the set of predictor variables. Here, ARD = Alzheimer’s-related dementia; nARD = non-Alzheimer’s-related dementia; SE = standard error; CI = confidence interval; MTA = medial temporal atrophy; GCA = global cortical atrophy.

Comparisons across individual dementia subtypes are shown in Table 3. Across individual subtypes, mixed dementia, frontotemporal dementia, and Parkinson’s disease dementia showed similar ages at diagnosis to Alzheimer’s disease, whereas vascular dementia was diagnosed approximately 2.9 years earlier. Pseudodementia, as a separate comparator group, presented substantially earlier, with a mean age of 53.2 ± 12.2 years. Hypertension was more prevalent in mixed dementia (58.8%) and vascular dementia (59.2%), while smoking was more frequent in vascular dementia (52.0%). Further, age- and sex-adjusted stepwise multivariable logistic regression analysis identified hypertension, OR 4.2 (95% CI 1.1–16.6), P = 0.04, and smoking, OR 13.1 (95% CI 1.6–103.3), P = 0.01, as factors associated with VD compared with ARD (S2 Table). Depression was most common in pseudodementia (63.3%) and frontotemporal dementia (60.0%). TSH levels were highest in frontotemporal dementia. Neuroimaging patterns also differed across subtypes: MTA scores were highest in mixed dementia, GCA scores were highest in frontotemporal dementia, and Fazekas scores were highest in vascular and mixed dementia (Table 3).

**Table 3:** Comparison of population characteristics between types of dementia.

	MD	VD	FTD	PDD	PD	^a^P value	^b^P value	^c^P value	^d^P value	^e^P value
Age, years (mean ± SD)	67.3 ± 10.4	64.8 ± 10.5	64.5 ± 11.0	67.8 ± 8.0	53.2 ± 12.2	0.73	0.01	0.08	0.99	<0.001
Men n (%)	58 (59.8%)	67 (68.4%)	22 (59.5%)	17 (70.8%)	27 (65.9%)	0.76	0.11	0.90	0.39	0.51
Residence Urban n (%)	25 (39.7%)	24 (30.8%)	13 (44.8%)	7 (36.8%)	16 (47.1%)	0.80	0.32	0.87	0.59	0.49
HTN n (%)	57 (58.8%)	58 (59.2%)	8 (21.6%)	13 (54.2%)	17 (41.5%)	0.03	0.03	0.003	0.48	0.51
Diabetes n (%)	35 (36.1%)	37 (37.8%)	7 (18.9%)	7 (29.2%)	13 (31.7%)	0.53	0.35	0.08	0.71	0.89
Depression n (%)	40 (52.6%)	27 (33.3%)	15 (60.0%)	8 (33.3%)	19 (63.3%)	0.74	<0.001	0.61	0.48	0.43
Smoking n (%)	7 (16.3%)	26 (52.0%)	7 (36.8%)	2 (12.5%)	3 (13.6%)	0.12	0.002	0.43	0.24	0.20
CKD n (%)	6 (100%)	6 (85.7%)	0 (0.0%)	2 (66.7%)	–	0.55	0.92	0.04	0.46	–
Familial dementia n (%)	12 (20.0%)	12 (20.3%)	2 (9.5%)	3 (21.4%)	3 (15.0%)	0.57	0.61	0.17	1.0	0.57
MRF (≥2RF) n (%)	53 (54.6%)	52 (53.1%)	10 (27.0%)	15 (62.5%)	16 (39.0%)	0.31	0.49	0.01	0.21	0.22
TSH (mean ± SD)	2.5 ± 1.7	5.6 ± 2.7	7.9 ± 20.6	1.9 ± 1.1	1.7 ± 0.9	0.08	0.07	<0.001	0.71	0.32
FT4 (mean ± SD)	1.6 ± 2.0	0.9 ± 0.2	2.1 ± 3.4	1.6 ± 1.2	2.0 ± 2.7	0.47	0.03	0.94	0.76	0.99
Koedam score (mean ± SD)	0.9 ± 0.6	0.8 ± 0.4	0.8 ± 0.9	0.5 ± 0.6	0.5 ± 0.6	0.20	0.005	0.11	0.005	<0.001
MTA score (mean ± SD)	2.0 ± 0.6	1.1 ± 0.5	1.1 ± 0.5	1.2 ± 0.5	1.2 ± 0.6	0.22	<0.001	<0.001	<0.001	<0.001
GCA score (mean ± SD)	1.1 ± 0.4	1.1 ± 0.4	1.7 ± 0.7	1.3 ± 0.6	1.1 ± 0.5	0.29	0.02	<0.001	0.64	0.10
Fazekas score (mean ± SD)	1.8 ± 0.5	1.9 ± 0.7	0.9 ± 0.4	1.3 ± 0.6	1.2 ± 0.7	<0.001	<0.001	0.54	0.06	0.08

ARD- Alzheimer’s-related Dementia is reference group; VD- Vascular Dementia, MD- Mixed Dementia, FTD- Frontotemporal Dementia, PD- Pseudo Dementia, PDD- Parkinson’s Disease Dementia, and SE- Standard Error. Independent t-test was used to compare continuous variables whereas Chi-square test, and Fisher Exact test used for categorical variables as appropriate. ^**a**^P value, ^**b**^P value, ^**c**^P value, ^**d**^P value, and ^**e**^P value represent statistical significance for MD, VD, FTD, PDD and PD, respectively. P values were not adjusted for multiple comparisons and are presented as exploratory analyses.

**Fig 1:**
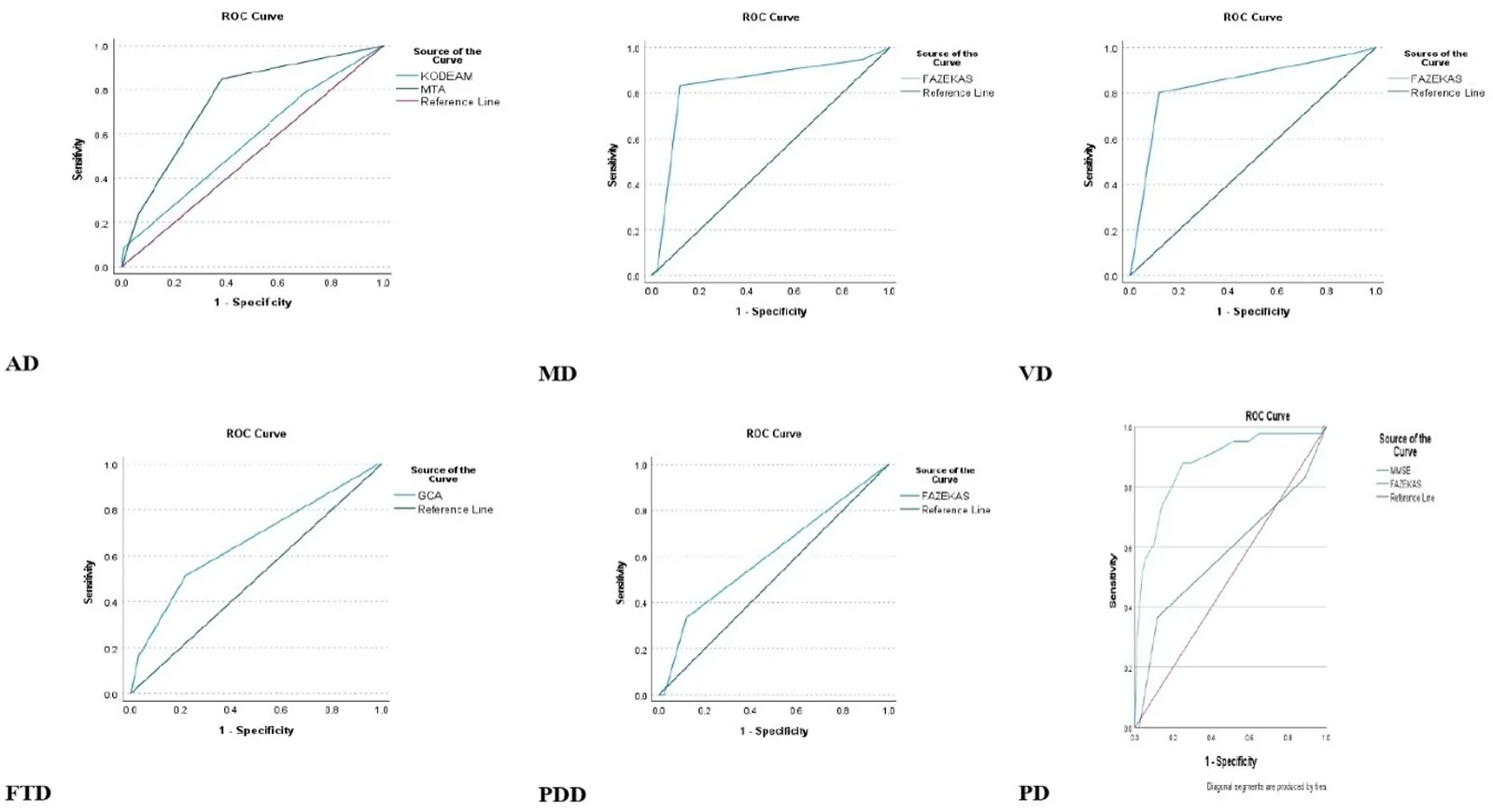
Receiver operating characteristic (ROC) curves for neuroimaging scores in differentiating dementia subtypes. AD = Alzheimer’s disease; VD = Vascular dementia; MD = Mixed dementia; FTD = Frontotemporal dementia; PDD = Parkinson’s disease dementia & PD = Pseudo dementia.

Receiver operating characteristic analyses demonstrated variable discriminatory performance of neuroimaging scales across dementia subtypes (Table 4). For differentiation of ARD from nARD, MTA showed good discriminatory ability (AUROC 0.752), whereas Koedam demonstrated poor discrimination (AUROC 0.570). Fazekas scores showed good discriminatory performance for mixed dementia (AUROC 0.836) and vascular dementia (AUROC 0.838). GCA showed moderate discriminatory ability for frontotemporal dementia (AUROC 0.659). Fig 1 illustrates the ROC curves for the evaluated neuroimaging scales (Fig 1). Further, no significant multicollinearity was identified among independent variables, and Little’s MCAR test indicated that missing data were consistent with a missing completely at random assumption.

**Table 4:** Discriminatory performance of neuroimaging scales across dementia subtypes.

Outcome (Subtype)	Scale	AUROC (95% CI)	Cut-off	Sensitivity (%)	Specificity (%)	PPV (%)	NPV (%)
ARD vs nARD	MTA	0.752 (0.71–0.79)	1.50	85.0	61.8	70.8	79.1
ARD vs nARD	Koedam	0.570 (0.53–0.61)	0.50	78.5	30.1	55.3	56.0
Mixed Dementia	Fazekas	0.836 (0.78–0.89)	1.50	83.3	88.2	66.7	94.9
Vascular Dementia	Fazekas	0.838 (0.79–0.89)	1.50	80.2	88.2	65.8	94.0
Frontotemporal Dementia	GCA	0.659 (0.56–0.76)	1.50	51.4	78.1	20.2	93.7

AUROC = area under the receiver operating characteristic curve; CI = confidence interval; PPV = positive predictive value; NPV = negative predictive value; ARD = Alzheimer’s-related dementia; nARD = non-Alzheimer’s-related dementia; MTA = medial temporal atrophy; GCA = global cortical atrophy. Optimal cut-off values were determined using the Youden index. Higher AUROC values indicate better discriminatory performance. Sensitivity, specificity, PPV, and NPV are reported as percentages. The 95% confidence intervals for the AUROC estimates were calculated using the nonparametric asymptotic method.

## Discussion

This study examined age at diagnosis of dementia, factors associated with dementia subtypes, and the discriminatory utility of neuroimaging scales in a Bangladeshi clinical population. A previous report from the CARED study platform examined the relationship between cognitive status and structural MRI atrophy scores in a small cohort restricted to Alzheimer’s disease [29]. The present study is distinct in including a larger Bangladeshi clinical cohort and extending the analysis to age at diagnosis, multiple dementia subtypes, vascular associations, and subtype-specific neuroimaging discrimination. This study found that Alzheimer’s-related dementia was diagnosed at a later age than non-Alzheimer’s-related dementia, while pseudodementia presented substantially earlier. Hypertension and smoking were associated with higher odds of vascular dementia. Neuroimaging scales demonstrated variable subtype-specific discriminatory utility. MTA, Fazekas, and GCA contributed differently across dementia subtypes.

Reported age at dementia diagnosis varies across Asia, Europe, and the United States and may be influenced by differences in genetics, lifestyle, healthcare access, referral patterns, and diagnostic practices [5,6]. Dementia in Asia usually begins in the late 60s to early 70s due to hypertension-related vascular issues [29,30], while in Europe and the US, it typically starts in the early to mid-70s, with Alzheimer’s disease as the most common subtype [31,32]. Non-Alzheimer’s dementia tends to have an earlier diagnosis than Alzheimer’s-related dementia potentially due to specific genetic mutations, protein accumulations, and cumulative vascular damage, which often arises earlier in individuals with cardiovascular risk factors, such as hypertension and smoking, consistent with our study findings [33–35]. In contrast, ARD usually develops later in life, as its primary risk factors are age and amyloid-beta pathology, which are more closely associated with the ageing process [11,12,19]. However, because this study assessed age at diagnosis rather than age at symptom onset, the observed difference may also reflect patterns of healthcare contact and case detection. Individuals with hypertension, smoking-related disease, or other vascular risk factors may receive more frequent medical assessments, increasing the likelihood that cognitive impairment is recognised and investigated earlier. Therefore, the younger age at diagnosis of dementia with vascular components should not be interpreted solely as evidence of earlier disease onset. The associations of hypertension and smoking with vascular dementia observed in this study align with established evidence linking vascular risk factors to cerebrovascular pathology and cognitive decline [36]. Hypertension contributes to small vessel disease and white matter damage [37], while smoking accelerates vascular injury and oxidative stress [38]. These findings also highlight the potential value of routine cognitive assessment in older adults with hypertension and other vascular risk factors, particularly in primary care, to support earlier recognition of cognitive impairment [39]. This may be particularly relevant in Bangladesh, where vascular risk factors are highly prevalent [40]. To our knowledge, this is among the first studies from Bangladesh to examine differences in age at diagnosis across dementia subtypes and factors associated with vascular dementia. The findings generate hypotheses regarding the potential importance of vascular risk-factor management and cognitive assessment, but require confirmation in larger, multicentre and population-based studies.

A key contribution of this study is the evaluation of established visual neuroimaging scales for differentiating dementia subtypes in a Bangladeshi clinical population. MTA demonstrated good discriminatory performance for ARD, supporting its relevance as a marker of medial temporal lobe involvement and hippocampal atrophy in Alzheimer’s disease [23,24]. However, medial temporal atrophy is not specific to Alzheimer’s disease and may also occur with normal ageing and other neurodegenerative disorders. Therefore, the MTA score should be interpreted as supportive evidence rather than as a standalone diagnostic marker. Fazekas scores showed the strongest discriminatory performance for vascular dementia and mixed dementia, consistent with the role of white matter hyperintensities and small vessel disease in vascular cognitive impairment [27]. This finding is also clinically coherent with the observed association of hypertension and smoking with vascular dementia in this cohort. Nevertheless, white matter changes are also common in older adults and may coexist with Alzheimer’s pathology. A higher Fazekas score therefore supports the presence of vascular brain injury but does not, by itself, establish vascular dementia. GCA showed moderate discriminatory ability for frontotemporal dementia. As global cortical atrophy is a non-specific measure, this finding may reflect overall cortical volume loss rather than the characteristic regional frontal or temporal patterns associated with frontotemporal dementia. Its low positive predictive value suggests limited ability to confirm the diagnosis. Although the negative predictive value was high, predictive values depend on disease prevalence and should be interpreted cautiously in this selected cohort, particularly given the relatively small number of patients with frontotemporal dementia. The Koedam score, despite being higher in ARD in baseline comparisons, showed limited discriminatory performance in ROC analysis, indicating that posterior atrophy alone may not reliably distinguish ARD from nARD in this setting. Overall, these findings suggest that structured MRI visual rating scales may provide useful adjunctive information when interpreted within a multidisciplinary diagnostic framework, but they should not be considered substitutes for clinical assessment.

The findings of this study should be interpreted in light of several limitations. Participants were recruited from a single tertiary-care centre, and referral patterns and differences in access to specialist assessment may have introduced selection bias and limited generalisability to the broader population. The relatively modest sample size, particularly within some dementia subtypes, may have limited statistical precision, as reflected by the wide confidence intervals around some odds-ratio estimates. Consequently, the findings should not be considered representative of the wider Bangladeshi population. Nevertheless, they provide important clinical insights into age at diagnosis, vascular risk-factor associations, and the potential adjunctive value of neuroimaging scales in dementia assessment within a resource-limited tertiary-care setting. The cross-sectional design limits causal inference, and the absence of longitudinal follow-up precludes assessment of disease progression and accurate determination of symptom onset. Longitudinal cohort studies would help clarify the timing of symptom onset and diagnosis. Additionally, formal dementia staging/severity measures were not consistently available and could not be analysed. Important socio-demographic factors such as education, occupation, and socioeconomic status were not available and may have influenced both age at diagnosis and clinical presentation, introducing residual confounding. Use of stepwise model selection may introduce instability and should be interpreted cautiously. No adjustment was made for multiple comparisons; therefore, some statistically significant findings, particularly in the pairwise subtype comparisons, may represent type I error. Although the neuroimaging scales demonstrated discriminatory utility, the imaging findings are not specific to individual dementia subtypes. ROC-derived cut-offs were developed and evaluated within the same dataset and were not externally validated, which may overestimate their performance. In addition, the AUROC confidence intervals were based on an asymptotic large-sample approximation and may be less reliable for dementia subtypes with small case numbers. Future studies could use bootstrap resampling in larger validation cohorts to assess the robustness of these estimates. Moreover, as several MRI atrophy scores increase with age, residual age confounding may remain despite adjusted analyses.

Despite these limitations, this study provides preliminary clinical evidence on dementia diagnosis among patients assessed at a tertiary referral centre in Bangladesh. The inclusion of a multidisciplinary diagnostic approach, incorporating neurological, psychiatric, and neuroimaging assessment, strengthened diagnostic accuracy and minimised diagnostic misclassification. Future research should focus on multicentre, population-based studies with longitudinal follow-up to improve generalisability and evaluate disease trajectories. Inclusion of detailed socio-demographic variables and external validation of neuroimaging-based diagnostic models will further enhance understanding and support the development of context-specific diagnostic and prevention strategies.

## Conclusions

Patients with Alzheimer’s-related dementia were diagnosed at an older age than those with non-Alzheimer’s-related dementia, while pseudodementia had the earliest age at diagnosis in this sample. Hypertension and smoking were associated with higher odds of vascular dementia compared with Alzheimer’s-related dementia. Neuroimaging scales demonstrated subtype-specific discriminatory utility, with medial temporal atrophy, Fazekas, and global cortical atrophy contributing differently to the differentiation of dementia subtypes. These findings suggest that structured MRI visual rating scales may provide useful adjunctive information when interpreted alongside multidisciplinary clinical assessment, but they should not be considered standalone diagnostic tools.

## Supporting information

S1 Table Baseline characteristics of study population (N = 658). (DOCX)

S2 Table Age- and gender adjusted stepwise multivariable logistic regression analysis predicting independent predictors of dementia. (DOCX)
