# Supplementary material for: Age at dementia diagnosis, subtype associations, and neuroimaging differentiation in Bangladesh: The CARED study
Source: PLoS One. 2026 Sep 25;21(9):e0359109. doi: 10.1371/journal.pone.0359109 (PMC13614553; doi:10.1371/journal.pone.0359109)
Supplement: Supplementary file 1 [file pone.0359109.s001.docx]

**Table S1:** Baseline characteristics of study population (N=658)

| Variables | | Men (n=397) | Women (n=261) | P value |
| --- | --- | --- | --- | --- |
| Age (years, mean ± SD) | | 66.2 ± 11.4 | 65.9 ± 11.3 | 0.73 |
| Residence | Rural (n%) | 99 (57.6%) | 73 (42.4%) | 0.65 |
|  | Urban (n%) | 122 (58.1%) | 87 (41.4%) |  |
|  | Town (n%) | 93 (62.4%) | 56 (37.6%) |  |
| HTN (n%) | | 186 (56.9%) | 141 (43.1%) | 0.12 |
| Diabetes (n%) | | 134 (60.9%) | 86 (39.1%) | 0.76 |
| Depression (n%) | | 152 (59.1%) | 105 (40.9%) | 0.26 |
| Smoking (n%) | | 96 (93.2%) | 6 (5.8%) | <0.001 |
| CKD (n%) | | 17 (56.7%) | 13 (43.3%) | 0.44 |
| Family H/O dementia (n%) | | 53 (60.9%) | 34 (39.1%) | 0.85 |
| MRF (≥2RF) (n%) | | 207 (63.1%) | 121 (36.9%) | 0.21 |
| Koedam score (mean ± SD) | | 0.9 ± 0.7 | 0.9 ± 0.8 | 0.82 |
| MTA score (mean ± SD) | | 1.7 ± 0.7 | 1.8 ± 0.8 | 0.33 |
| GCA score (mean ± SD) | | 1.2 ± 0.5 | 1.2 ± 0.6 | 0.94 |
| Fazekas score (mean ± SD) | | 1.3 ± 0.6 | 1.3 ± 0.7 | 0.87 |

**Note:** Data are presented as mean ± standard deviation (SD) for continuous variables and number (%) for categorical variables. Chi-square test or Fisher’s exact test was used for categorical variables, and independent samples t-test was used for continuous variables, as appropriate. A two-tailed P value < 0.05 was considered statistically significant. HTN = hypertension; CKD = chronic kidney disease; Family H/O = family history of dementia; MRF (≥2RF) = multiple risk factors (two or more risk factors); MTA = medial temporal atrophy; GCA = global cortical atrophy.
