# Supplementary material for: Age at dementia diagnosis, subtype associations, and neuroimaging differentiation in Bangladesh: The CARED study
Source: PLoS One. 2026 Sep 25;21(9):e0359109. doi: 10.1371/journal.pone.0359109 (PMC13614553; doi:10.1371/journal.pone.0359109)
Supplement: Supplementary file 2 [file pone.0359109.s002.docx]

**Table S2:** Age and gender adjusted stepwise multivariable logistic regression analysis predicting independent predictors of dementia.

| Variables | **ARD vs nARD** | | **MD** | | **VD** | | **FTD** | | **PDD** | | **PD** | |
| --- | --- | --- | --- | --- | --- | --- | --- | --- | --- | --- | --- | --- |
|  | OR (95% CI) | P value | OR (95% CI) | P value | OR (95% CI) | P value | OR (95% CI) | P value | OR (95% CI) | P value | OR (95% CI) | P value |
| Age |  |  | 0.9 (0.9–1.0) | 0.03 | 0.9 (0.9–1.0) | <0.001 |  |  |  |  | 0.9 (0.9–1.0) | 0.002 |
| HTN |  |  |  |  | 4.2 (1.1–16.6) | 0.04 |  |  |  |  |  |  |
| TSH |  |  |  |  |  |  |  |  |  |  |  |  |
| Smoking |  |  |  |  | 13.1 (1.6–103.3) | 0.01 |  |  |  |  |  |  |
| Koedam | 1.5 (1.0–2.2) | 0.03 |  |  |  |  |  |  | 0.4 (0.1–0.8) | 0.01 | 0.4 (0.2–0.9) | 0.02 |
| MTA | 7.9 (4.5–13.9) | <0.001 |  |  |  |  | 0.1 (0.0–0.4) | <0.001 | 0.1 (0.1–0.2) | <0.001 | 0.2 (0.1–0.3) | <0.001 |
| GCA |  |  |  |  |  |  | 4.2 (1.3–13.4) | 0.01 |  |  |  |  |
| Fazekas | 0.18 (0.1–0.3) | <0.001 | 8.8 (4.7–16.3) | <0.001 | 10.6 (6.5–17.4) | <0.001 |  |  | 3.0 (1.4–6.5) | 0.005 | 4.4 (2.0–9.7) | <0.001 |
| **Note:** Age- and sex-adjusted stepwise multivariable logistic regression models were used to identify independent factors associated with each dementia subtype. Odds ratios (ORs) with 95% confidence intervals (CIs) are presented for variables retained in the final models. Variables initially entered into the models included age, sex, hypertension (HTN), diabetes mellitus (DM), chronic kidney disease (CKD), residence, depression, multiple risk factors (MRF), smoking, thyroid-stimulating hormone (TSH), free thyroxine (FT4), family history (H/O) of dementia, Koedam score, medial temporal atrophy (MTA) score, global cortical atrophy (GCA) score, and Fazekas score. The ARD versus nARD model compared Alzheimer’s-related dementia with all non-Alzheimer’s-related dementia diagnoses, with nARD as the reference category. For the individual subtype models, ARD was used as the reference category. Blank cells indicate variables not retained in the final model for that subtype. A two-tailed P value < 0.05 was considered statistically significant. ARD = Alzheimer’s-related dementia; MD = mixed dementia; VD = vascular dementia; FTD = frontotemporal dementia; PDD = Parkinson’s disease dementia; PD = pseudodementia. | | | | | | | | | | | | |
