# Supplementary material for: Age at dementia diagnosis, subtype associations, and neuroimaging differentiation in Bangladesh: The CARED study
Source: PLoS One. 2026 Sep 25;21(9):e0359109. doi: 10.1371/journal.pone.0359109 (PMC13614553; doi:10.1371/journal.pone.0359109)
Supplement: Supplementary file 3 [file pone.0359109.s004.docx]

**Response to Reviewers**

Dear Editor

We thank you for giving our manuscript careful consideration and thank the Reviewers for helping us to improve it.

Please find attached a revised manuscript and a line-by-line response to the Reviewer’s queries.

We trust this manuscript now meets with your approval and look forward to hearing from you.

Yours sincerely,

Dr Redoy Ranjan PhD

Assistant Editor, PLOS One
Department of Cardiac Surgery, Bangabandhu Sheikh Mujib Medical University,
Dhaka, Bangladesh
Email: [redoy_ranjan@bsmmu.edu.bd](mailto:redoy_ranjan@bsmmu.edu.bd)

**Response to Reviewer 1**

**Comment 1:** Given the relatively limited sample size of 658 patients, several statements should be toned down, particularly regarding the generalisability of the findings to the wider Bangladeshi population.

**Response:** We thank the reviewer for this important comment. We agree that the single-centre setting and relatively modest sample size, particularly within some dementia subtypes, limit the generalisability of the findings. We have therefore strengthened the Limitations section to clarify that the results should not be considered representative of the wider Bangladeshi population.

**Revision made:** We toned down the interpretation and generalisability of the findings in the Discussion (lines 312–314), expanded the limitations concerning the single-centre setting, modest sample size, statistical precision, referral bias, and representativeness of the wider Bangladeshi population (lines 335–349), and clarified the clinical relevance of the findings within a tertiary-care setting (line 363).

**Comment 2:** A greater discussion of the potential meaning of the neuroradiological scores should be presented, beyond their statistical significance.

**Response:** We thank the reviewer for this important comment. We agree that the clinical interpretation and limitations of the neuroimaging scales should be discussed beyond their statistical performance. We have therefore expanded the Discussion to explain the potential clinical meaning, non-specificity, and appropriate interpretation of the MTA, Fazekas, GCA, and Koedam scores. We have also emphasised that these scales should be used as supportive components of multidisciplinary assessment rather than as standalone diagnostic markers.

**Revision made:** We expanded the Discussion (lines 312–334) to clarify that medial temporal atrophy may support a diagnosis of Alzheimer’s-related dementia but is also seen with normal ageing and other neurodegenerative disorders. We clarified that higher Fazekas scores support the presence of vascular brain injury but do not independently establish vascular dementia, as white matter changes may occur in older adults and coexist with Alzheimer’s pathology. We also explained that global cortical atrophy is a non-specific measure and that its predictive values should be interpreted cautiously, particularly given the relatively small frontotemporal dementia subgroup. Finally, we clarified that the Koedam score showed limited ability to distinguish Alzheimer’s-related from non-Alzheimer’s-related dementia. The Limitations section was also revised (lines 351–360) to state that the neuroimaging scales are based on visual ratings, are not specific to individual dementia subtypes, and that the internally derived ROC cut-offs require external validation.

**Comment 3:** The earlier diagnosis of dementia with vascular components may reflect greater or earlier medical attention among people with prevalent cardiovascular risk factors, and this possibility should be discussed.

**Response:** We thank the reviewer for this important observation. We agree that the younger age at diagnosis among patients with dementia with vascular components may not necessarily indicate earlier disease onset. More frequent healthcare contact among individuals with hypertension, smoking-related disease, or other vascular risk factors may lead to earlier recognition and investigation of cognitive impairment.

**Revision made:** We revised the Discussion (lines 290–296) to clarify that the study assessed age at diagnosis rather than age at symptom onset and that differences in healthcare contact and case detection may partly explain the earlier diagnosis of dementia with vascular components. We also revised the Limitations section (lines 335–360) to state that the cross-sectional design and absence of longitudinal follow-up prevented accurate determination of symptom onset, and that longitudinal cohort studies would help clarify the timing of symptom onset and diagnosis.

**Comment 4:** The relevance of screening cognitive function in individuals with hypertension and other vascular risk factors should be mentioned.

**Response:** We thank the reviewer for this helpful suggestion. We have added the potential benefit of cognitive assessment in older adults with hypertension.

**Revision made:** We added a statement to the Discussion (lines 300–308) highlighting the potential value of routine cognitive assessment in older adults with hypertension and other vascular risk factors, particularly in primary care with the citation.

**Response to Reviewer 2:**

**Comment 1:** On page 11, R² = 0.03 has been interpreted as modest to moderate. However, although it is statistically significant, it explains only 3% of the variation. The model therefore explained only a small proportion of the variability in age at diagnosis.

**Response:** We thank the reviewer for identifying this inaccurate interpretation. We have made the correction accordingly.

**Revision made:** We revised the Results section (line 208-209) to replace the description “modest to moderate” with the statement that the model explained only a small proportion of the variability in age at diagnosis.

**Comment 2:** PLOS generally prefers results to be reported as OR (95% CI), P. This format has not been used consistently. For example, hypertension and smoking were reported without their corresponding 95% confidence intervals.

**Response:** We thank the reviewer for highlighting this inconsistency. We have revised the manuscript accordingly and checked the reporting of results for consistency.

**Revision made:** We revised the Abstract and Results sections (lines 61–62 and 228–230) to report the associations of hypertension and smoking with vascular dementia using the format OR (95% CI), P. Statistical results, spacing, decimal places, and confidence-interval formatting were standardised throughout the manuscript and supplementary material.

**Comment 3:** The ROC analysis looks excellent. However, please clarify whether the ROC confidence intervals were obtained using the DeLong method or bootstrapping.

**Response:** We thank the reviewer for this helpful comment. The 95% confidence intervals for the AUROC estimates were calculated using the nonparametric asymptotic method implemented in SPSS. We have now clarified this in the manuscript.

**Revision made:** We added a statement to the Statistical analysis section (lines 160–162) and the footnote to Table 4 (lines 259–260), specifying that the 95% confidence intervals for the AUROC estimates were calculated using the nonparametric asymptotic method in SPSS. We also revised the Limitations section (lines 357–360) to acknowledge that this large-sample approximation may provide less precise estimates for subtypes with small sample sizes and that future studies could assess the robustness of the findings using bootstrap resampling in larger validation cohorts.

**Comment 4:** Were P values adjusted for multiple comparisons? If not, please mention this in the Limitations section.

**Response:** We thank the reviewer for raising this point. No adjustment was made for multiple comparisons because the subtype analyses were exploratory. We agree that this may increase the possibility of type I error and have clarified this in the manuscript.

**Revision made:** We added a statement to the Statistical analysis section (line 170) specifying that no adjustment was made for multiple comparisons because the subtype analyses were exploratory. We also added a note to Table 3 (line 241-242) stating that the P values were unadjusted and are presented as exploratory analyses. The Limitations section was revised (line 351-353) to acknowledge that some statistically significant findings, particularly in the pairwise subtype comparisons, may represent type I error.

**Comment 5:** Use consistent spacing throughout the manuscript, for example, write P = 0.03 instead of P=0.03.

**Response:** We thank the reviewer for identifying this formatting inconsistency. We have standardised the spacing and presentation of statistical notation throughout the manuscript and supplementary material.

**Revision made:** We revised the manuscript to ensure consistent spacing around statistical symbols. P values are now reported as P = 0.03 or P < 0.001 in the narrative text, and consistent spacing has been applied to confidence intervals, R² values, comparison symbols, and mean ± SD results throughout.

**Comment 6:** In Table 1, for TSH and FT4, consider writing the variable name as mean ± SD and check for consistency throughout the manuscript.

**Response:** We thank the reviewer for identifying this inconsistency. We have revised the manuscript and made corrections accordingly.

**Revision made:** We revised Table 1 to label TSH and FT4 as mean ± SD. We also revised rest of the manuscript to consistently present the continuous variables.

**Comment 7:** A sample-size calculation has not been stated. If participants were recruited consecutively, please clarify this in the Methods.

**Response:** All eligible patients presenting during the predefined study period were included consecutively. As the study used consecutive recruitment rather than a predetermined target sample, no a priori sample-size calculation was performed.

**Revision made:** We revised the Study design and setting subsection (lines 112–115) to clarify that all eligible individuals presenting during the study period were consecutively included and that no a priori sample-size calculation was performed.

We are grateful to the Reviewers for helping us to improve our manuscript and trust that it now meets his/her approval.

With thanks and regards,

Dr Redoy Ranjan PhD
