# Supplementary material for: Age at dementia diagnosis, subtype associations, and neuroimaging differentiation in Bangladesh: The CARED study
Source: PLoS One. 2026 Sep 25;21(9):e0359109. doi: 10.1371/journal.pone.0359109 (PMC13614553; doi:10.1371/journal.pone.0359109)
Supplement: Peer review history

# Peer review: Decision Letter 0

**DOI:** 10.1371/journal.pone.0359109.r001

**Authors:** Ioannis Liampas

27 Jul 2026

Dear Dr. Ranjan,

Thank you for submitting your manuscript to PLOS ONE. After careful consideration, we feel that it has merit but does not fully meet PLOS ONE’s publication criteria as it currently stands. Therefore, we invite you to submit a revised version of the manuscript that addresses the points raised during the review process.

Please submit your revised manuscript by Sep 25 2026 11:59PM. If you will need more time than this to complete your revisions, please reply to this message or contact the journal office at plosone@plos.org. When you're ready to submit your revision, log on to https://www.editorialmanager.com/pone/ and select the 'Submissions Needing Revision' folder to locate your manuscript file.

- A letter that responds to each point raised by the academic editor and reviewer(s). You should upload this letter as a separate file labeled 'Response to Reviewers'.
- A marked-up copy of your manuscript that highlights changes made to the original version. You should upload this as a separate file labeled 'Revised Manuscript with Track Changes'.
- An unmarked version of your revised paper without tracked changes. You should upload this as a separate file labeled 'Manuscript'.

If you would like to make changes to your financial disclosure, please include your updated statement in your cover letter. Guidelines for resubmitting your figure files are available below the reviewer comments at the end of this letter.

If applicable, we recommend that you deposit your laboratory protocols in protocols.io to enhance the reproducibility of your results. Protocols.io assigns your protocol its own identifier (DOI) so that it can be cited independently in the future. For instructions see: https://journals.plos.org/plosone/s/submission-guidelines#loc-laboratory-protocols. Additionally, PLOS ONE offers an option for publishing peer-reviewed Lab Protocol articles, which describe protocols hosted on protocols.io. Read more information on sharing protocols at https://plos.org/protocols?utm_medium=editorial-email&utm_source=authorletters&utm_campaign=protocols.

As the corresponding author, your ORCID iD is verified in the submission system and will appear in the published article. PLOS supports the use of ORCID, and we encourage all coauthors to register for an ORCID iD and use it as well. Please encourage your coauthors to verify their ORCID iD within the submission system before final acceptance, as unverified ORCID iDs will not appear in the published article. *Only*  the individual author can complete the verification step; PLOS staff *cannot*  verify ORCID iDs on behalf of authors.

We look forward to receiving your revised manuscript.

Kind regards,

Ioannis Liampas, MD. PhD

Academic Editor

PLOS One

**Journal Requirements:**

When submitting your revision, we need you to address these additional requirements.

1. Please ensure that your manuscript meets PLOS ONE's style requirements, including those for file naming. The PLOS ONE style templates can be found at

https://journals.plos.org/plosone/s/file?id=wjVg/PLOSOne_formatting_sample_main_body.pdf and

https://journals.plos.org/plosone/s/file?id=ba62/PLOSOne_formatting_sample_title_authors_affiliations.pdf

2. Please describe in your methods section how capacity to provide consent was determined for the participants in this study. Please also state whether your ethics committee or IRB approved this consent procedure. If you did not assess capacity to consent please briefly outline why this was not necessary in this case.

3. In the online submission form you indicate that your data is not available for proprietary reasons and have provided a contact point for accessing this data. Please note that your current contact point is a co-author on this manuscript. According to our Data Policy, the contact point must not be an author on the manuscript and must be an institutional contact, ideally not an individual. Please revise your data statement to a non-author institutional point of contact, such as a data access or ethics committee, and send this to us via return email. Please also include contact information for the third party organization, and please include the full citation of where the data can be found.

4. Your ethics statement should only appear in the Methods section of your manuscript. If your ethics statement is written in any section besides the Methods, please delete it from any other section.

5. Please include captions for your Supporting Information files at the end of your manuscript, and update any in-text citations to match accordingly. Please see our Supporting Information guidelines for more information: http://journals.plos.org/plosone/s/supporting-information.

6. If the reviewer comments include a recommendation to cite specific previously published works, please review and evaluate these publications to determine whether they are relevant and should be cited. There is no requirement to cite these works unless the editor has indicated otherwise.

[Note: HTML markup is below. Please do not edit.]

Reviewers' comments:

Reviewer's Responses to Questions

**Comments to the Author**

1. Is the manuscript technically sound, and do the data support the conclusions?

Reviewer #1: Yes

Reviewer #2: Yes

**********

2. Has the statistical analysis been performed appropriately and rigorously?

Reviewer #1: Yes

Reviewer #2: Yes

**********

3. Have the authors made all data underlying the findings in their manuscript fully available?

The PLOS Data policy

Reviewer #1: No

Reviewer #2: No

**********

4. Is the manuscript presented in an intelligible fashion and written in standard English?

Reviewer #1: Yes

Reviewer #2: Yes

**********

Reviewer #1: The manuscript deals with an interesting topic

Given the relatively limited population size (658 patietns) many sentence should be toned down, with specific regards to the generalazibility of the findings to the whole Bangladesh population

A greater selection in the potential meaning of the neuroradiological scores adopted should be presented and siscussed - over and above the statistical significance of the p value

It should be considered and sicussed as the earlier diagnosis of dementia with vascular components may be due to a greater and ealier medical attention to subjects with prevalent CV risk factors.

Within this context, it should be mentioned the relevance of screening for cognitive function in subjects with hypertension and other vascular risk factors (PMID: 33273363)

Reviewer #2: 1. On page 11, R² = 0.03 has been interpreted as modest to moderate. However, although it is statistically significant, it explains only 3% of the variation. The model therefore explained only a small proportion of the variability in age at diagnosis.

2. PLOS generally prefers the reporting style OR (95% CI), P, which has not been used consistently throughout the manuscript. For example, on page 12, HTN (OR 4.2, P = 0.04) and smoking (OR 3.1, P = 0.01) have been reported without the corresponding 95% confidence intervals.

3. Your ROC analysis looks excellent. However, I was just wondering whether the ROC confidence intervals were obtained using the DeLong method or bootstrap. Kindly add one sentence in the Methodology describing this.

4. Were p-values adjusted for multiple comparisons? If not, then please mention this in the Limitations section.

5. Suggestion: Use consistent spacing throughout the manuscript (e.g., write P = 0.03 instead of P=0.03).

6. In Table 1, for TSH and FT4, consider writing the variable name as Mean ± SD and check for consistency throughout the manuscript.

7. A sample size calculation has not been stated anywhere, although it is generally expected. If the participants were recruited consecutively, then please state this in one sentence in the Methods section.

**********

what does this mean?). If published, this will include your full peer review and any attached files.

If you choose “no”, your identity will remain anonymous but your review may still be made public.

**Do you want your identity to be public for this peer review?**  For information about this choice, including consent withdrawal, please see our Privacy Policy

Reviewer #1: No

Reviewer #2: **Yes:** Arifa Tabassum

**********

[NOTE: If reviewer comments were submitted as an attachment file, they will be attached to this email and accessible via the submission site. Please log into your account, locate the manuscript record, and check for the action link "View Attachments". If this link does not appear, there are no attachment files.]

To ensure your figures meet our technical requirements, please review our figure guidelines: https://journals.plos.org/plosone/s/figures

You may also use PLOS’s free figure tool, NAAS, to help you prepare publication quality figures: https://journals.plos.org/plosone/s/figures#loc-tools-for-figure-preparation.

NAAS will assess whether your figures meet our technical requirements by comparing each figure against our figure specifications.

# Author comment: Author response to Decision Letter 1

**DOI:** 10.1371/journal.pone.0359109.r002

19 Aug 2026

Response to editorial comments:

1. We have revised the data availability statement as requested and have provided a non-author institutional contact, specifically a data access officer from the NINS&H ethics committee, Bangladesh.

2. We have removed all duplicate files. We apologise for any inconvenience.

3. We have removed the ethics statement from the acknowledgements section as advised.

Response to Reviewer 1

Comment 1: Given the relatively limited sample size of 658 patients, several statements should be toned down, particularly regarding the generalisability of the findings to the wider Bangladeshi population.

Response: We thank the reviewer for this important comment. We agree that the single-centre setting and relatively modest sample size, particularly within some dementia subtypes, limit the generalisability of the findings. We have therefore strengthened the Limitations section to clarify that the results should not be considered representative of the wider Bangladeshi population.

Revision made: We toned down the interpretation and generalisability of the findings in the Discussion (lines 312–314), expanded the limitations concerning the single-centre setting, modest sample size, statistical precision, referral bias, and representativeness of the wider Bangladeshi population (lines 335–349), and clarified the clinical relevance of the findings within a tertiary-care setting (line 363).

Comment 2: A greater discussion of the potential meaning of the neuroradiological scores should be presented, beyond their statistical significance.

Response: We thank the reviewer for this important comment. We agree that the clinical interpretation and limitations of the neuroimaging scales should be discussed beyond their statistical performance. We have therefore expanded the Discussion to explain the potential clinical meaning, non-specificity, and appropriate interpretation of the MTA, Fazekas, GCA, and Koedam scores. We have also emphasised that these scales should be used as supportive components of multidisciplinary assessment rather than as standalone diagnostic markers.

Revision made: We expanded the Discussion (lines 312–334) to clarify that medial temporal atrophy may support a diagnosis of Alzheimer’s-related dementia but is also seen with normal ageing and other neurodegenerative disorders. We clarified that higher Fazekas scores support the presence of vascular brain injury but do not independently establish vascular dementia, as white matter changes may occur in older adults and coexist with Alzheimer’s pathology. We also explained that global cortical atrophy is a non-specific measure and that its predictive values should be interpreted cautiously, particularly given the relatively small frontotemporal dementia subgroup. Finally, we clarified that the Koedam score showed limited ability to distinguish Alzheimer’s-related from non-Alzheimer’s-related dementia. The Limitations section was also revised (lines 351–360) to state that the neuroimaging scales are based on visual ratings, are not specific to individual dementia subtypes, and that the internally derived ROC cut-offs require external validation.

Comment 3: The earlier diagnosis of dementia with vascular components may reflect greater or earlier medical attention among people with prevalent cardiovascular risk factors, and this possibility should be discussed.

Response: We thank the reviewer for this important observation. We agree that the younger age at diagnosis among patients with dementia with vascular components may not necessarily indicate earlier disease onset. More frequent healthcare contact among individuals with hypertension, smoking-related disease, or other vascular risk factors may lead to earlier recognition and investigation of cognitive impairment.

Revision made: We revised the Discussion (lines 290–296) to clarify that the study assessed age at diagnosis rather than age at symptom onset and that differences in healthcare contact and case detection may partly explain the earlier diagnosis of dementia with vascular components. We also revised the Limitations section (lines 335–360) to state that the cross-sectional design and absence of longitudinal follow-up prevented accurate determination of symptom onset, and that longitudinal cohort studies would help clarify the timing of symptom onset and diagnosis.

Comment 4: The relevance of screening cognitive function in individuals with hypertension and other vascular risk factors should be mentioned.

Response: We thank the reviewer for this helpful suggestion. We have added the potential benefit of cognitive assessment in older adults with hypertension.

Revision made: We added a statement to the Discussion (lines 300–308) highlighting the potential value of routine cognitive assessment in older adults with hypertension and other vascular risk factors, particularly in primary care with the citation.

Response to Reviewer 2:

Comment 1: On page 11, R² = 0.03 has been interpreted as modest to moderate. However, although it is statistically significant, it explains only 3% of the variation. The model therefore explained only a small proportion of the variability in age at diagnosis.

Response: We thank the reviewer for identifying this inaccurate interpretation. We have made the correction accordingly.

Revision made: We revised the Results section (line 208-209) to replace the description “modest to moderate” with the statement that the model explained only a small proportion of the variability in age at diagnosis.

Comment 2: PLOS generally prefers results to be reported as OR (95% CI), P. This format has not been used consistently. For example, hypertension and smoking were reported without their corresponding 95% confidence intervals.

Response: We thank the reviewer for highlighting this inconsistency. We have revised the manuscript accordingly and checked the reporting of results for consistency.

Revision made: We revised the Abstract and Results sections (lines 61–62 and 228–230) to report the associations of hypertension and smoking with vascular dementia using the format OR (95% CI), P. Statistical results, spacing, decimal places, and confidence-interval formatting were standardised throughout the manuscript and supplementary material.

Comment 3: The ROC analysis looks excellent. However, please clarify whether the ROC confidence intervals were obtained using the DeLong method or bootstrapping.

Response: We thank the reviewer for this helpful comment. The 95% confidence intervals for the AUROC estimates were calculated using the nonparametric asymptotic method implemented in SPSS. We have now clarified this in the manuscript.

Revision made: We added a statement to the Statistical analysis section (lines 160–162) and the footnote to Table 4 (lines 259–260), specifying that the 95% confidence intervals for the AUROC estimates were calculated using the nonparametric asymptotic method in SPSS. We also revised the Limitations section (lines 357–360) to acknowledge that this large-sample approximation may provide less precise estimates for subtypes with small sample sizes and that future studies could assess the robustness of the findings using bootstrap resampling in larger validation cohorts.

Comment 4: Were P values adjusted for multiple comparisons? If not, please mention this in the Limitations section.

Response: We thank the reviewer for raising this point. No adjustment was made for multiple comparisons because the subtype analyses were exploratory. We agree that this may increase the possibility of type I error and have clarified this in the manuscript.

Revision made: We added a statement to the Statistical analysis section (line 170) specifying that no adjustment was made for multiple comparisons because the subtype analyses were exploratory. We also added a note to Table 3 (line 241-242) stating that the P values were unadjusted and are presented as exploratory analyses. The Limitations section was revised (line 351-353) to acknowledge that some statistically significant findings, particularly in the pairwise subtype comparisons, may represent type I error.

Comment 5: Use consistent spacing throughout the manuscript, for example, write P = 0.03 instead of P=0.03.

Response: We thank the reviewer for identifying this formatting inconsistency. We have standardised the spacing and presentation of statistical notation throughout the manuscript and supplementary material.

Revision made: We revised the manuscript to ensure consistent spacing around statistical symbols. P values are now reported as P = 0.03 or P < 0.001 in the narrative text, and consistent spacing has been applied to confidence intervals, R² values, comparison symbols, and mean ± SD results throughout.

Comment 6: In Table 1, for TSH and FT4, consider writing the variable name as mean ± SD and check for consistency throughout the manuscript.

Response: We thank the reviewer for identifying this inconsistency. We have revised the manuscript and made corrections accordingly.

Revision made: We revised Table 1 to label TSH and FT4 as mean ± SD. We also revised rest of the manuscript to consistently present the continuous variables.

Comment 7: A sample-size calculation has not been stated. If participants were recruited consecutively, please clarify this in the Methods.

Response: All eligible patients presenting during the predefined study period were included consecutively. As the study used consecutive recruitment rather than a predetermined target sample, no a priori sample-size calculation was performed.

Revision made: We revised the Study design and setting subsection (lines 112–115) to clarify that all eligible individuals presenting during the study period were consecutively included and that no a priori sample-size calculation was performed.

We are grateful to the Reviewers for helping us to improve our manuscript and trust that it now meets his/her approval.

With thanks and regards,

Dr Redoy Ranjan PhD

Attachment Submitted filename: Response.docx

# Peer review: Decision Letter 1

**DOI:** 10.1371/journal.pone.0359109.r003

**Authors:** Ioannis Liampas

9 Sep 2026

<p>Age at dementia diagnosis, subtype associations, and neuroimaging differentiation in Bangladesh: The CARED study

PONE-D-26-29498R1

Dear Dr. Ranjan,

We’re pleased to inform you that your manuscript has been judged scientifically suitable for publication and will be formally accepted for publication once it meets all outstanding technical requirements.

Within one week, you’ll receive an e-mail detailing the required amendments. When these have been addressed, you’ll receive a formal acceptance letter and your manuscript will be scheduled for publication.

An invoice will be generated when your article is formally accepted. Please note, if your institution has a publishing partnership with PLOS and your article meets the relevant criteria, all or part of your publication costs will be covered. Please make sure your user information is up-to-date by logging into Editorial Manager at Editorial Manager® and clicking the ‘Update My Information' link at the top of the page. For questions related to billing, please contact billing support.

If your institution or institutions have a press office, please notify them about your upcoming paper to help maximize its impact. If they’ll be preparing press materials, please inform our press team as soon as possible -- no later than 48 hours after receiving the formal acceptance. Your manuscript will remain under strict press embargo until 2 pm Eastern Time on the date of publication. For more information, please contact onepress@plos.org.

Kind regards,

Ioannis Liampas, MD. PhD

Academic Editor

PLOS One

Additional Editor Comments (optional):

Reviewers' comments:

Reviewer's Responses to Questions

**Comments to the Author**

Reviewer #1: All comments have been addressed

Reviewer #2: All comments have been addressed

**********

2. Is the manuscript technically sound, and do the data support the conclusions?

Reviewer #1: Yes

Reviewer #2: Yes

**********

3. Has the statistical analysis been performed appropriately and rigorously?

Reviewer #1: Yes

Reviewer #2: Yes

**********

4. Have the authors made all data underlying the findings in their manuscript fully available?

The PLOS Data policy

Reviewer #1: Yes

Reviewer #2: Yes

**********

5. Is the manuscript presented in an intelligible fashion and written in standard English?

Reviewer #1: Yes

Reviewer #2: Yes

**********

Reviewer #1: (No Response)

Reviewer #2: The statistical reporting format is understandable; however, it could be presented more clearly and consistently in accordance with PLOS ONE conventions. The manuscript currently reports the estimate as “OR 13.1 (95% CI 1.6-103.3).” I recommend revising it to “OR = 13.1 (95% CI: 1.6-103.3)” and applying this format consistently throughout the manuscript.

**********

what does this mean?). If published, this will include your full peer review and any attached files.

If you choose “no”, your identity will remain anonymous but your review may still be made public.

**Do you want your identity to be public for this peer review?**  For information about this choice, including consent withdrawal, please see our Privacy Policy

Reviewer #1: No

Reviewer #2: **Yes:** Arifa Tabassum

**********

# Editor report: Acceptance letter

**DOI:** 10.1371/journal.pone.0359109.r004

**Authors:** Ioannis Liampas

PONE-D-26-29498R1

PLOS One

Dear Dr. Ranjan,

I'm pleased to inform you that your manuscript has been deemed suitable for publication in PLOS One. Congratulations! Your manuscript is now being handed over to our production team.

At this stage, our production department will prepare your paper for publication. This includes ensuring the following:

* All references, tables, and figures are properly cited

* All relevant supporting information is included in the manuscript submission,

* There are no issues that prevent the paper from being properly typeset

You will receive further instructions from the production team, including instructions on how to review your proof when it is ready. Please keep in mind that we are working through a large volume of accepted articles, so please give us a few days to review your paper and let you know the next and final steps.

Lastly, if your institution or institutions have a press office, please let them know about your upcoming paper now to help maximize its impact. If they'll be preparing press materials, please inform our press team within the next 48 hours. Your manuscript will remain under strict press embargo until 2 pm Eastern Time on the date of publication. For more information, please contact onepress@plos.org.

You will receive an invoice from PLOS for your publication fee after your manuscript has reached the completed accept phase. If you receive an email requesting payment before acceptance or for any other service, this may be a phishing scheme. Learn how to identify phishing emails and protect your accounts at https://explore.plos.org/phishing.

If we can help with anything else, please email us at customercare@plos.org.

Thank you for submitting your work to PLOS One and supporting open access.

Kind regards,

PLOS One Editorial Office Staff

on behalf of

Dr. Ioannis Liampas

Academic Editor

PLOS One
